# Studies on the Possibility of Introducing New Materials into Reproductive Biotechnology and a New Type of Catheter for Embryo Transfer

**DOI:** 10.3390/ani16060905

**Published:** 2026-03-13

**Authors:** Jarosław Wieczorek, Ewa Stodolak-Zych, Krzysztof Okoń, Jurij Koseniuk, Magdalena Bryła, Małgorzata Kotula-Balak, Jacek Jura, Katarzyna Poniedziałek-Kempny, Iwona Rajska, Katarzyna Soból, Aleksandra Lasoń–Kubarek, Izabela Krakowska, Grzegorz Fraś

**Affiliations:** 1 Faculty of Veterinary Medicine, University of Agriculture in Krakow, Mickiewicza 24/28, 30-059 Krakow, Poland; malgorzata.kotula-balak@urk.edu.pl (M.K.-B.); izabela.krakowska@urk.edu.pl (I.K.); grzegorz.fras@urk.edu.pl (G.F.); 2 Department of Biomaterials, Faculty of Materials Science and Ceramics, University of Science and Technology, Mickiewicza 30, 30-059 Krakow, Poland; stodolak@agh.edu.pl; 3 Chair of Pathomorphology, Collegium Medicum, Jagiellonian University, Grzegorzecka 16, 30-526 Krakow, Poland; k.okon@uj.edu.pl; 4 Artvimed Centre for Reproductive Medicine, IVF Laboratory Czyzowka 14, 30-526 Krakow, Poland; jurij.koseniuk@artvimed.pl; 5 Department of Reproductive Biotechnology and Cryoconservation, National Research Institute of Animal Production, Krakowska 1, 30-322 Krakow, Poland; magdalena.bryla@iz.edu.pl (M.B.); jacek.jura@iz.edu.pl (J.J.); katarzyna.sobol@iz.edu.pl (K.S.); 6 Department of Pig Breeding, National Research Institute of Animal Production, Krakowska 1, 30-322 Krakow, Poland; katarzyna.poniedzialek@iz.edu.pl; 7 Department of Animal Molecular Biology, National Research Institute of Animal Production, Krakowska 1, 30-322 Krakow, Poland; iwona.rajska@iz.edu.pl; 8 Podgorskie Veterinary Center, Na Dolach 6, 30-704 Krakow, Poland; a.lason311@gmail.com

**Keywords:** biomaterial, embryos, transfer, catheter

## Abstract

The use of seven biomaterials was examined in the study: polyethylene (PE), polypropylene (PP), polyurethane (PU), teflon (PTFE), polycaprolactone (PCL), polylactide (PLLA), and a copolymer of poly L-lactide and dibutyryl chitin (PLLA/DBC). The use of these materials has not been discussed in the context of animal reproduction so far. Due to the specific nature of the reproductive system and the high sensitivity of reproductive cells, at the outset the biocompatibility and cytotoxicity of the materials were tested in somatic cell and embryo cultures. Additionally, the material properties of the catheters were determined in terms of the roughness of the internal and external surfaces, the stability of the shape of the catheters, their elasticity, durability, and ductility. Finally, clinical testing of the developed catheters was performed in laparoscopic transplantation of embryos into the uterine tubes of the sows. Significant toxicity of PTFE, PCL and PLLA/DBC towards the embryos was indicated in the cytotoxicity testing. In the materials testing, numerous flaws of the PP, PLLA, and PLLA/DBC catheters were indicated. In the final stage, catheters were developed using PE and PU. In clinical testing, these latter catheters exhibited high effectiveness in transferring embryos. Among the seven biomaterials tested, only polyethylene and polyurethane exhibited high biocompatibility and the material properties mentioned above. There is thus good indication for the introduction of these catheters for embryo transfer in animal reproduction biotechnology.

## Simple Summary

Reproductive biotechnology has advanced in recent years, but embryo transfer has not advanced as much; methods and catheters from 30 years ago are still being used. Therefore, an attempt was made to introduce innovative catheters made of new, previously unused biomaterials. This study investigated the possibility of introducing new, previously unused materials into reproduction and the development of catheters for embryo transplantation. Seven biomaterials used in surgery with confirmed potential for use in human and veterinary medicine were tested. Comprehensive testing of these materials was conducted, assessing their biocompatibility with respect to somatic cells and embryos, and conducting biomaterial studies. Biomaterial testing identified materials with appropriate properties, including strength, elasticity, extensibility, and surface structure. Of the seven biomaterials tested, only polyethylene and polyurethane demonstrated high biocompatibility and the aforementioned material properties. Therefore, there are good grounds for implementing these catheters for embryo transfer in animal reproductive biotechnology.

## 1. Introduction

Natural and synthetic biomaterials with high compatibility regarding living tissues are used widely and routinely in medicine and veterinary sciences. However, data from the literature indicate that the least common usage of biomaterials is in the reproductive sciences and in gynecology, of humans and in particular of animals. In human medicine, methods have been described for the treatment of dysfunction of the female and male reproductive system using biomaterials and for the use of biomaterials in assisted reproductive technology (ART), mainly in embryo transfer [1]. Traditional amelioration methods mainly involve hormone therapy, surgical methods, and organ transplant [2]. These methods have a limited impact on regaining function and fertility due to their lack of regenerative ability and the risk of serious rejection of reproductive organs and tissues [3]. The use of biomaterials in veterinary sciences in reproductive therapy is significantly limited and rarely seen [4]. In the design of biomaterials intended for treatment of the reproductive system or in assisted reproductive systems, attention is paid to two main aspects. These are low cytotoxicity and high biocompatibility with organs and tissues in the body, and the appropriate material properties, such as elasticity, durability, surface structure, and degradation. The desired material properties must ensure the least possible trauma to the tissues in the course of the procedure and the best possible fit to the specific structure of the organs and the physiological environment [5]. Regarding the aspect of toxicity and biocompatibility, the reproductive cells may be considerably more sensitive than somatic cells [6]. For this reason, additional verification is required of the toxic properties of the materials in contact with oocytes, embryos and spermatozoa. A further relevant factor is the material properties of the materials, such as their ability to bind with proteins, hormones, and cells and also their coating with tissues [7]. Materials with these characteristics can significantly accelerate the regeneration of damaged tissue or exhibit quite opposite effects [8]. Biomaterials used for the regeneration of tissue in situ or in artificial assisted reproductive technologies (ART) when compared to materials used in somatic tissues must exhibit greater elasticity, durability, and an appropriate surface structure, and not cause injury, bleeding or inflammatory states while the reproductive tract is being exposed to manipulation [9,10,11]. There is a serious lack of appropriate materials in animal reproductive biotechnology. For example, various embryo transfer methods are used with animals based on highly diverse types of catheters made from various materials. Different types of catheters are used for transferring embryos and reproductive biotechnology, including human ones, and in many cases these are adapted or modified catheters for artificial insemination such as the Folley Tomcat, cannula-type intravenous catheters, or numerous proprietary designs [11,12,13,14,15,16,17,18,19].

According to guidelines and principles for human ET, ET catheters should ideally be biocompatible, non-toxic to embryos, soft enough to prevent damage to the lining of the fallopian tube, uterus, and cervix, yet flexible enough to extend to the desired length into the uterine lumen or fallopian tube naturally. They should be as thin as possible and allow for embryo transfer in a very short time (up to 2–4 min) and with minimal media (approximately 20 μL) [20]. Despite numerous reported embryo transfer protocols in humans and animals, the characteristics of a good catheter have not been clearly defined, and no consensus has been reached on standards for ET practice. One possible explanation is that the success of the ET procedure depends on many factors, several of which are difficult to standardize and therefore difficult to study. These include: operator experience, degree of difficulty of the procedure, technique of loading the embryo catheter (air bubbles, characteristics of the culture medium, volume of fluid), injection pressure and speed, duration of ET, presence of blood or mucus in the genital tract or catheter, type of catheter, clinical condition and age of recipients, as well as different variants of the method [9,21].

Significant progress in the development and implementation of the above methods in animal reproduction occurred at the turn of the 20th and 21st centuries, when laparoscopic methods for obtaining oocytes, embryos and embryo transfer were introduced with great intensity, followed by non-invasive methods of embryo transfer. Because of this variety of transfer techniques used, the effectiveness of transfer among animals is highly varied, ranging from a dozen or so to more than 90% [13,14,16,22,23,24,25]. And due to the limited application and low availability of advanced biomaterials in reproductive biotechnology of animals, progress is needed in this area in terms of material engineering. It is essential to introduce new types of catheters for embryo transfer, based not only on known and proven materials, but also to design and introduce new types of biomaterials into biotechnology and animal reproduction. These efforts are an important area of research with great potential for development and deserve greater attention. Moreover, due to the complex structure and function of reproductive organs and gametes among animals, it has not been possible so far to create completely reliable biomaterials which would promote structural regeneration and the regaining of the function of reproductive glands. At the same time, considerable progress in material engineering would allow for the introduction of new solutions, not used until now. The aim of this paper was to examine the feasibility of introducing new embryo transfer catheters and introducing previously unused materials for animal reproduction and gynecology.

## 2. Materials and Methods

The study was approved by the 2nd Local Ethics Commission in Kraków (Poland) (no. 791/2010; 30 September 2010 and 582/2008; 25 September 2008). The study was conducted in compliance with conditions outlined in Directive 2010/63/EU of the European Parliament and Council dated 22 September 2010 on the protection of animals used for scientific purposes.

### 2.1. Experimental Setup

The study covered 7 biomaterials: polycaprolactone (PCL) (polycaprolactone, Merck KGaA, Darmstadt, Germany), polylactide (PLLA) (Polylactic acid, Ingeo^®^ 3041D, NatureWorks, Minnetonka, MN, USA), polypropylene (PP) (Propylene, Irganox^®^ 1035, BASF, Ludwigshafen, Germany), polyethylene (PE) (Polyethylene, LDPE, BASF, Ludwigshafen, Germany), teflon (PTFE) (Polytetrafluoroethylene, Merck KGaA, Darmstadt, Germany), copolymer of poly L-lactide and dibutyryl chitin (PLA/DBC, Technical University of Łódź, Łódź, Poland), and polyurethane (PU) (Polyurethane, Merck KGaA, Darmstadt, Germany). The study was conducted in two stages. The first stage concerned the verification of the biocompatibility of the materials with regard to embryos and determination of their material properties. Biocompatibility verification involved a comparison of biotoxicity of the materials in embryo or somatic cell cultures, with direct contact of the material with the cells. Cytotoxicity testing was conducted in compliance with the EN-ISO 10933-5 norm [26]. Material studies concerned the possibility of creating catheters with the desired qualities (appropriate length, stability of shape, and smoothness of surface) and adequate elasticity and durability. The aim was to create catheters with a length of 20–25 cm and a minimum external diameter of 1.0–1.5 mm (matching the diameter of the clear passage through the uterine tube). The second stage involved the clinical verification of the utility of the catheters created in which embryos were transferred in animals. The transplantation efficiency and developmental potential of transplanted embryos were examined at this stage.

Verification of biocompatibility in embryo or somatic cell cultures on biomaterials (PCL, PLA, PLA/DBC, PP, PE, PTFE, PU).

Discs were cut out of the materials of the same size as the wells in the cultivation plates, and a 5-day culture was performed with somatic cells or embryos. The control group for these cultures comprised embryos or somatic cells cultured without contact with the biomaterial under the same conditions.

### 2.2. Somatic Cell Culture

Osteoblast cells of the h.FOB 1.19 line (hFOB 1.19 human fetal osteoblastic cell line, CRL-3602, American Type Culture Collection, Manassas, VA, USA) was cultured in an OGM Bulle Kit (OGM Bulle Kit, Cat. No. CC-3207, Lonza, Basel, Switzerland) growth medium, in an atmosphere of 5% CO_2_ at a temperature of 37 °C for 5 days. Cells from the 3–4 passage were used for the study. The cell solution was obtained by adding 5% trypsin with EDTA (Ethylenediaminetetraacetic acid, Lonza, Basel, Switzerland). After being centrifuged, the cells were taken to a concentration of 1.5 × 10^4^ cells/mL, following which 1 mL of the solution was placed in the wells of a 24-well culture plate (Nunclon, Merck KGaA, Darmstadt, Germany) which held the sterile discs of the studied materials. After 5 days of culture, the viability of the cells was assessed.

### 2.3. Embryo Culture and Assessment of Embryo Developmental Potential

Culture was conducted in embryo culture plates in vitro for 5 days, in an NCSU-23 medium [27] at a temperature of 39 °C and an atmosphere of 5% CO_2_. Assessment of the embryos during culture was performed daily under a stereoscopic microscope (100× enlargement), in a laminar flow cabinet, at a temperature of approx. 30 °C (magnification of over 50–100×) (Nikon SMZ 10A, Tokyo, Japan). All blastocysts obtained were tested with the TUNEL method (Terminal deoxynucleotidyl transferase dUTP nick end labelling, In Situ Cell Death Detection Kit, Roche, Mannheim, Germany), assessing the number of cells in the blastocysts and the apoptosis index. The number of stained cells (nuclei) in the blastocysts was counted under an Eclipse E600 epifluorescence microscope (Nikon, Tokyo, Japan), using a filter with a wavelength of 358–461 nm, with blue fluorescence of all cell nuclei. The number of apoptotic nuclei was counted using a filter with a wavelength of 520 nm, with green fluorescence of apoptotic cells. Assessment of nuclear DNA fragmentation was based on a comparison of the total nucleus number and the number of positively stained nuclei indicating DNA fragmentation (apoptotic index).

On the basis of the results of cytotoxicity and material testing, materials were identified for further clinical testing.

Testing of materials in terms of their potential for use in the creation of catheters and study of the properties of the catheters

Prototype tubes were made from the studied biomaterials. Matrixes were created by extruding tubes with a diameter of 5.0 mm and a length of 10 cm. Next, by making use of the thermoplastic properties of polymers, the tubes were pulled onto a metal stylet with a diameter of 0.8 mm and a length of 25 cm. The catheters were manufactured using the proprietary patent: “Method of manufacturing catheters” [28]. In the study of the material properties of the biomaterials, three parameters were assessed: 1. The structure of the tubes’ surface, 2. The stability of the shape of the tubes, and 3. The mechanical properties of the catheters. The tests were performed on the basis of ISO standards applicable to medical materials and devices [29,30].

### 2.4. Study of the Surface Structure

The morphology of the external and internal surfaces of the catheters was assessed (Ultra high-definition Scanning Electron Microscope, SEM, Nova NanoSEM 200, FEI Europe B.V., Eindhoven, The Netherlands). Standard surface roughness parameters were recorded: Ra—arithmetic mean deviation of the roughness profile, Rt—maximum height between the highest peak and the lowest valley, Rz—height of the roughness profile assessed according to ten points (5 highest peaks and 5 lowest valleys). Each tube of 40 mm length was subjected to 5 measurements. 5 tubes of each type of material were studied.

### 2.5. Study of the Stability of Tube Shape

The stability of the shape of the tubes was assessed via an assessment of the geometry, external and internal diameter, thickness of walls, and deviation from these values (Digital microscope, KEYENCE International, Mechelen, Belgium).

### 2.6. Study of Mechanical Properties

The ductile properties of the tubes, their elasticity and durability were assessed. The mechanical testing determined: deformation—ε (extension at maximum force: FmaX), tensile strength—Rm, stiffness (Young’s modulus—E), and fracture toughness (work of destruction)—W. The following study parameters were applied: measurement base—a tube with a length of 40 mm, speed of stretching 40 mm/min, (ZWICK 1435 universal testing machine, ZWICK, Ulm, Germany).

### 2.7. Clinical Testing—Assessment of the Biocompatibility of the Catheters in In Vivo Tests

Assessment involved determination of the effectiveness of embryo transfer to the uterine tubes (percentage of pregnancies obtained, number of young born) and study of the trauma inflicted upon the uterine tubes by histological assessment of the uterine tubes after embryo transfer. At this stage, the use of catheters with an internal diameter of 1.0 mm was assumed. Embryos were transferred at the 2–4 blastomere development stage using a laparoscopic method described previously [13] to 38 recipients (using the PE catheter n = 24 and the PU catheter n = 14). Thirty recipients (PE n = 20, PU n = 10) were left for diagnosis of pregnancy, while the remaining 8 recipients (PE n = 4, PU n = 4) were euthanised 5 days after transfer, the transferred embryos were flushed, and the uterine tubes were removed for histopathological analysis. The uteri from these pigs were flushed for blastocyst collection. The control group was blastocysts obtained from donors and cultured in vitro as previously described. The developmental potential of the blastocysts was assessed using the TUNEL method, as previously described. The recipients and donors were subjected to routine synchronization of the osetrus cycle. Superovulation was also induced in the donors according to a regimen described previously [13]. At the development stage of 2–4 blastomeres, the embryos were collected surgically according to a protocol described previously [13].

The recipients were under general infusion anesthesia. The animals were premedicated with azaperone at a dosage of 3 mg/kg i.m. (Stresnil, Jannsen, Neuss, Germany). General anesthesia was induced by administering 2% xylazine at a dosage of 1–2 mg/kg i.v. (Xylapan 2%, Vetoquinol, Gorzów Wielkopolski, Poland) and 10% ketamine at a dosage of 5–10 mg/kg i.v. (Ketamine 10%, Biovet, Puławy, Poland). The embryos were introduced into the uterine tubes via uterine puncture. The camera of the endoscope was positioned centrally between the 2nd and 3rd pair of teats on the left side, with the left grip situated in the navel and the right grip between the 4th and 5th pair of teats, 3–5 cm laterally from the central line. The catheter was introduced into the abdominal cavity at the level of the 5th pair of teats, laterally from the central line. The transplantation kit comprised a guide in the form of a needle adapted to the external diameter of the catheter and an elastic tube with a diameter of 1 mm and a length of 20 cm. After stabilization of the uterine tube with the guide, uterine puncture was performed and the catheter with embryos was introduced into the passage of the uterine tube to a depth of 3–5 cm. The embryos were placed at the tip of the catheter (1–2 cm) with a minimal volume of liquid (approx. 20 µL). The embryos were deposited by injection into the passage of the uterine tube. They were deposited either in the right or left uterine tube. After deposition, the catheter, grips, and trocars were removed in reverse order from that in which they were applied. After removal of the trocars, single simple PGA stitches were applied to the skin (PGA 1, DKO117PG, Yavo, Bełchatów, Poland). The peritoneum and muscles did not require stitches. Pregnancy was diagnosed between the 28th and 31st day after the procedure by USG. The percentage of pregnancies obtained, the number of young born, and the number of young weaned were estimated.

### 2.8. Assessment of the Developmental Potential of Embryos After Transplantation

The transferred embryos were incubated in the uterus for 5 days. After this time, the recipients were euthanized. Uteri and oviducts were collected for analysis. The blastocysts were obtained by washing the uterus. The developmental potential of the washed blastocysts was assessed using the TUNEL method, as described above. The collected oviducts were assessed histologically.

### 2.9. Histological Assessment of the Oviducts After Embryo Transfer

Uterine tubes were removed from 8 recipients on the 5th day after embryo transfer (PE n = 4, PU n = 4). Both uterine tubes were removed from the sows for analysis, one which had been subjected to the procedure and the second as a control. The uterine tubes were immersed in a 10% solution of formaldehyde, and then cut with a microtome cutting tool into cross-sections with a length of 2–3 mm. The samples were placed in histology cassettes and encased in paraffin. Cuttings with a thickness of 2–3 µm were prepared from the paraffin blocks, and these were placed on base slides and stained using the haematoxylin and eosin Shandon, method in an automated slide stainer (Shandon Varistain Gemini, Thermo Fisher Scientific, Waltham, MA, USA).

### 2.10. Statistical Analysis

Statistical analysis was performed using Statistica 13.3 software (Tibco Statistica, Krakow, Poland). One-way analysis of variance for independent samples was performed using the ANOVA test, with a significance level of *p* < 0.05. Variables were treated as independent samples. Statistically significant differences between groups were determined using Student’s *t* test, and consistency between results was measured using the standard deviation (mean ± SD).

## 3. Results

### 3.1. Verification of the Biocompatibility of Materials in Somatic Cell and Embryo Cultures

The results are presented in Table 1. The results of biological testing confirmed the fact that the biomaterials are safe for somatic cells (the survival rate of osteoblasts of the h.FOB 1.19 cell line in contact with the surface of the instrument was approx. 89% for PE and 90% for PP, while for the control group this number was 100%). None of the materials qualified for testing are cytotoxic. Taking into account the fact that somatic cells are organisms with much less sensitivity than reproductive cells, in the subsequent stage of the study, the biocompatibility of the contacting materials was studied with regard to embryos obtained from sows.

After being placed on PTFE, PCL, and PLLA/DBC substrates, all cells were frozen at an early stage of development, and neither morulae nor blastocysts developed. The remaining materials demonstrated a high level of biocompatibility. A higher percentage of morulae and blastocysts was obtained in the PU, PP, PLLA, and PE groups than in the control group. Statistically higher percentages of morulae and blastocysts were obtained in the PP and PLLA groups (differences A and B). However, cultures on PP and PLLA materials demonstrated significantly lower cell counts in blastocysts (differences C–F) and significantly lower apoptotic cell counts and apoptotic indexes compared to the PU, PE, and control groups (differences G–J). In cultures on PP and PLLA materials, blastocysts did not fully develop, as was the case with cultures on PU, PE, and the control group. Despite high survival rates, PP and PLLA materials could have hindered embryo development, reducing their developmental potential. Of the materials tested, PU and PE had the best properties, the highest biocompatibility, and the greatest developmental potential.

### 3.2. Assessment of Surface Structure

**Figure 1:**
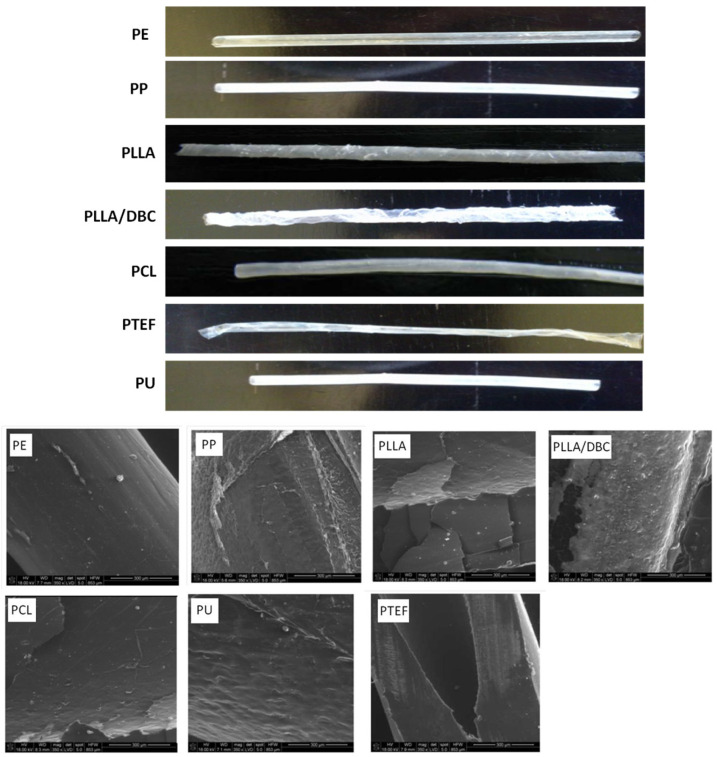
Surface structure of the tubes. Macroscopic assessment of the external morphology of the catheter surface and appearance of the catheters under SEM (scanning electron microscope) analysis.

The results are presented in Table 2 and Figure 1. In the deviation from the roughness profile (Ra), the deviations formed a sequence of PE < PU < PCL < PP < PTFE < PLLA/DBC < PLLA. The lowest deviation was exhibited by the PE and PU tubes (significant statistical differences with regard to PP, PLLA, PLLA/DBC, PCL, and PTFE). Considerable differences are apparent in the deviation of the structures in the PP, PLLA and PLLA/DBC. There were no statistical differences in the deviation of the roughness profile between the external and internal surfaces. In the assessment of the height of the roughness profile (Rz), results similar to those above were obtained. The lowest profile was exhibited by the PE and PU catheters. A high profile was exhibited by the catheters made from PP, PLLA, and PLLA/DBC (significant statistical differences with to PE, PU). There were no statistical differences between the external and internal surfaces. In SEM analysis, neither deep surface damage, perforation nor discontinuities of the PE, PU, PCL or PTFE catheters were observed. It was concluded that minor cavities on the external surface of these catheters did not affect their safety of use. Roughness was identified on the surface of the PP catheters, but the continuity of the tubes was maintained. In the case of PLLA and PLLA/DBC catheters, lack of surface homogeneity (roughness) and numerous discontinuities of the material were observed.

### 3.3. Study of the Stability of Catheter Shape

The results are presented in Table 3. Depending on the material used, tubes were obtained with an external diameter of 0.9–4.32 mm (the wall diameter was dependent on the biomaterial chosen: PE, PP, PLLA, PLLA/DBC, PCL, PU, or PTFE). The best possibilities for forming and ease of removal from moulds were demonstrated by the PE catheters, which delivered tubes with an external diameter of less than 1.0 mm while maintaining an internal open diameter of approx. 0.8 mm, low wall thickness, and stability of shape, together with the possibility of creating tubes of any length (greater than 40 cm). In the case of PE tubes, in all the studied parameters statistically significant differences were shown with regard to the remaining groups. Similar parameters to those seen with PE were observed in the case of tubes made from PCL, PU, and PTFE. Nonetheless, in the case of tubes made from these materials, the stability of tube shape was not confirmed. In the case of tubes made from PP, it was shown to be possible to form tubes of any length and of a stable shape; however, it was not shown to be possible to create tubes with a small diameter, allowing for introduction into the uterine tube (average tube diameter 1.83 mm). In the case of PLLA and PLLA/DBC tubes, it was not shown to be possible to form tubes longer than 10 cm and with the desired material structure. Tubes made from this material were of a considerably larger external and internal diameter and exhibited considerable variation in shape.

### 3.4. Study of Mechanical Properties

**Figure 2:**
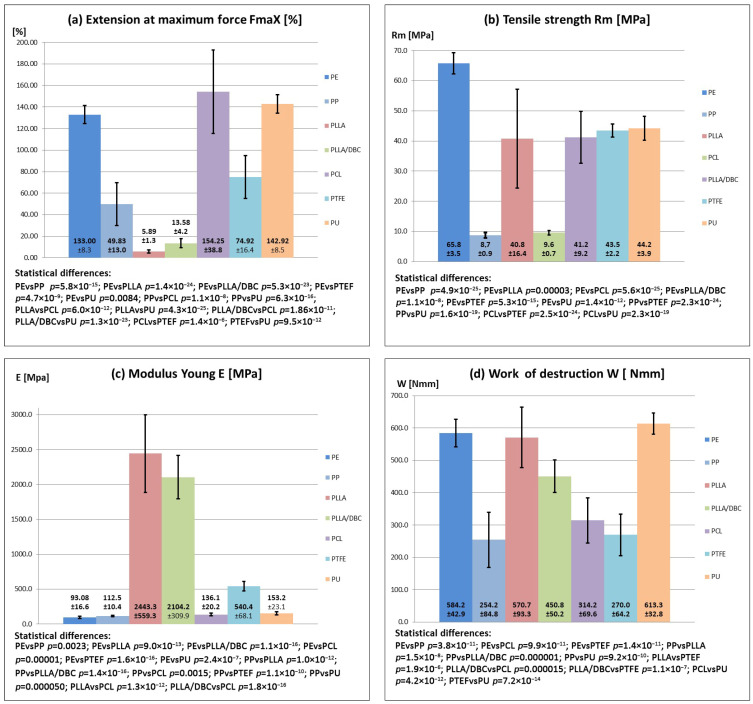
Mechanical properties, resistance to deformation, ductility, elasticity, and fracture toughness of tubes made from the studied materials, (**a**) extension at maximum force: FmaX (%); (**b**) tensile strength (MPa); (**c**) Young’s modulus (MPa); (**d**) (work of destruction (Nmm).

Changes in durability (Extension at maximum force) occurred according to the sequence: PCL < PU < PE < PTFE < PP < PLLA/DBC < PLLA (statistical differences are presented in Figure 2a). The greatest extensibility of the tubes was observed in tubes made of PCL < PU and PE. Tensile strength occurred according to the sequence: PE < PU < PTFE < PLLA/DBC < PLLA < PTFE < PP (statistical differences are marked on the graph in Figure 2b). The highest tensile strength was observed in tubes made of PE, PU, and PTFE. Changes in stiffness occurred (Young’s modulus) in the sequence PE < PP < PCL < PU < PTFE < PLLA/DBC < PLLA (statistical differences are presented in Figure 2c). The greatest flexibility was observed in tubes made of PE, PP, and PCL. The durability and resistance to damage of the tubes followed the sequence PP < PTFE < PCL < PLLA/DBC < PLLA < PE < PU. (Statistical differences are presented in Figure 2d). PLLA, PE, and PU tubes exhibited the greatest resistance to damage. Of the tubes tested, PE, PU, and PCL exhibited desirable material properties. Tubes made of these materials demonstrated adequate durability, ductility, and elasticity. The remaining materials, PP, PLLA, PTFE, and PLLA/DBC, did not demonstrate adequate mechanical properties.

### 3.5. Transplantation of Embryos

Embryo transfer was performed using catheters made from PE (n = 20) and PU (n = 10) with a diameter of 1.00 mm. The results obtained are presented in Table 4.

962 embryos were obtained from 80 donors. 833 embryos were transplanted to achieve pregnancy and offspring. On average, 28.0 (PE) and 27.3 (PU) embryos were transferred to one recipient. Pregnancy rates of 75.0% (PE) and 70.0% (PU) were obtained. On average, 10.4 (PE) and 10.0 (PU) young were born to each recipient. 86% and 85% of the young born were successfully weaned, respectively. There were no statistical differences between the groups (PE and PU). The remaining 124 embryos were transplanted to 8 recipients (PE = 64/4, PU = 60/4) from whom oviducts were obtained for histological examination and blastocysts were obtained to assess the developmental potential of the embryos. Five embryos were used as in vivo controls for testing developmental potential. The embryos transplanted with catheters demonstrated high developmental potential. The resulting blastocysts had numerous cell nuclei with a low apoptotic index. No differences were observed either in the number of nuclei and in the apoptotic index; both clinical (PE, PU) and control groups and had a similar number of nuclei in blastocysts and a very similar apoptotic index.

### 3.6. Histology of Uterine Tubes

**Figure 3:**
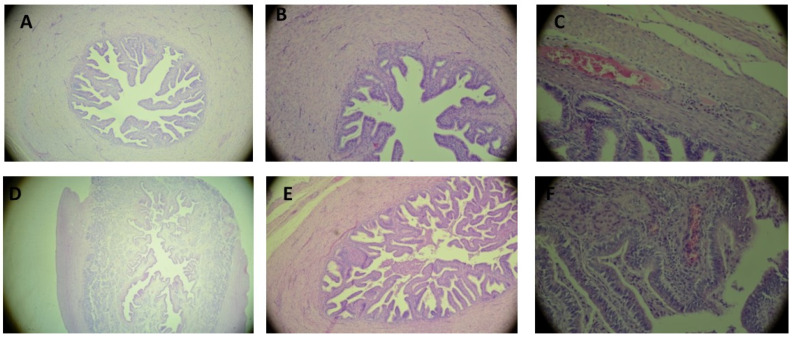
Histological analysis of the uterine tubes: (**A**–**C**)—uterine tube after transplantation using a catheter made from PE; (**D**–**F**)—control uterine tube. (**A**)—uterine tube within the physiological norm, visible individual granulocytes (PE; 100×); (**B**)—slight concentrations of purulence in the vicinity of the uterine tube (PE; 200×); (**C**)—sparse granulocytic infiltration in the uterine tube wall and concentrations of granulocytes in the clear diameter of the vessels (PE; 400×); (**D**)—structure of the uterine tube maintained, concentration of shed epithelial cells in the passage of the uterine tube (100×); (**E**)—minor inflammatory infiltration (Control; 200×); (**F**)—very minor site of inflammatory infiltration (Control; 400×).

The results are presented in Table 5 and Figure 3. After transfer of the embryos using PE and PU catheters from the 8 collected uterine tube pairs, very similar changes were observed. The clear passage of all uterine tubes was fully maintained. In one uterine tube from each group, a slight degree of inflammatory infiltration (1st degree) was observed. One PE recipient had moderate granulocytic infiltrate in the fallopian tube wall (statistical difference A). In one uterine tube treated with PE, slight purulent lesions were observed. Additionally, in one of the control uterine tubes, a small amount of inflammatory infiltration was observed. In terms of structural changes, intensification of inflammatory infiltration, and maintenance of the clear passage of the uterine tubes, considerable similarity was shown between the experimental groups and the control group.

## 4. Discussion

For many years, natural, synthetic, and hybrid biomaterials [2,31] have been used with a high degree of effectiveness in the treatment of animals. Despite such widespread application of biomaterials, the possibilities of their use in the biotechnology of animal reproduction have not been considered until now. While in human medicine significant attention is paid to the type and quality of catheters used, this issue has not been considered so far in veterinary medicine and animal reproduction. Embryo transfer is one of the most important steps leading to success in the long and arduous process of assisted reproduction. For example, embryo transfer (ET) is the final and one of the most important stages of in vitro fertilization (IVF), embryo culture (IVM), cloning, obtaining chimeras, transgenic animals, ex situ conservation of animal genetic resources, or commercial MOET methods used in various species [15,32]. Therefore, the undertaken research, for the first time, focused on the properties of materials used in the production of catheters and finished products in the form of prototype catheters for embryo transfer in animals. The choice of catheter for embryo transfer depends on many material, biological, and medical factors, e.g., operator preferences and the risk of complications [33]. Before introducing new materials to reproductive practices, it is essential to verify material properties of the catheters developed in terms of their biocompatibility with regard to embryos and to confirm their functionality in laboratory tests and then in vivo on animals. The current study covered new-generation materials such as PCL, PLLA, and PLA/DBC, PTFE and materials which have been known for years such as PE, PU and PP. There is a wealth of information confirming the broad possibilities for application of these materials and all of them are routinely used in clinical [34,35] and experimental [36,37,38,39] medicine. Over the course of many years of comparison of catheters for the transfer of embryos, attention has been drawn to their material properties, primarily concerning the surface structure, elasticity and susceptibility to deformation [33,40,41]. The use of catheters with inappropriate material quality may lead to failure of the transfer and the occurrence of post-op complications [42,43]. Stiff catheters facilitate the introduction of the embryos into the uterus, in particular in cases where the transfer is difficult due to, for example, anatomical anomalies. This, however, involves the risk of damaging the endometrium or uterine tubes, of bleeding, of stimulating the uterus or uterine tubes to contractions, and of hindering implantation [21]. Conversely, there is information suggesting that these complications do not impact the effectiveness of the transfer and may even increase the effectiveness of transfer [44]. Catheters with an inappropriate surface structure may also cause complications, mainly mechanical damage to the endometrium leading to disruption of its continuity and also hindering proper positioning of the casing [45]. In material terms, in the transfer of embryos in humans and animals, it is assumed that the catheters used should be of the least possible thickness, resistant to breaking, and elastic enough that the catheter can flex along the reproductive tract without damaging the endometrium or uterine tubes [46]. In the study presented here, an attempt was made to find an elastic, durable catheter with a diameter of 1.0 mm. It was assumed that a smaller catheter with the least smooth surface possible would allow for the introduction of the catheter without damaging the mucus membranes of the uterine tubes. As it has been shown, the appropriate material qualities were demonstrated by tubes made of PE, PU, PCL and PTFE. In turn, PP, PLLA and PLLA/DBC were eliminated due to the impossibility of achieving tubes with the appropriate characteristics. This resulted from the difficulty in forming catheters with a diameter of less than 1.5 mm from these latter materials and from their considerable stiffness and roughness of surface.

As has been pointed out earlier, the selection of materials was also guided by their low toxicity and proven biocompatibility with somatic cells [47,48,49,50,51,52]. For this reason, their biocompatibility with embryos was also assumed. This hypothesis, however, required direct verification. In toxicity studies, highly diverse results were obtained. The very high toxicity of polycaprolactone (PCL), dibutyryl chitin (PLA/DBC), and Teflon (PTFE) with regard to embryos was demonstrated and thus the use of these biomaterials in reproductive biotechnology was eliminated as a possibility, as was their use as a material for the production of catheters for the transfer of embryos. On the other hand, the possibility of using polyethylene (PE), polypropylene (PP), polylactide (PLLA), and polyurethane (PU) was demonstrated. An interesting observation was made, indicating that some materials with confirmed biocompatibility are highly toxic to embryos. On the other hand, embryos are significantly more sensitive to toxic factors than somatic cells. Biocompatibility of selected materials (PE and PU) was also confirmed using the TUNEL method. The occurrence of apoptosis in the pre-implantation embryonic stages was a crucial part of the assessment of the quality of the embryos obtained [53]. The phenomenon of apoptosis occurs in stages of embryonic development earlier than the blastocyst as a response to abnormalities associated with embryonic development [54,55]. The occurrence of the phenomenon of apoptosis in embryos produced in vitro is impacted by factors such as: exogenic sperm enzymes, increasing glutathione levels in the oocytes, the level of reactive oxygen species, and suboptimal conditions for oocyte and embryo culture [56,57,58]. Disturbances in embryonic development cause changes in embryo morphology visible mainly in the fragmentation of the cytoplasm. This anomaly which appears in the early stages of cell division impacts both the further development of the embryo and the effectiveness of insemination, the percentage of blastocysts obtained, and the number of cell nuclei [59]. The results of the study presented here indicate the diverse impact of materials on the development potential of embryos. In the case of PE and PU, a high embryo development potential was observed, while in the case of PP and PLLA this development potential was lower. Polyethylene and polyurethane are highly biocompatible, which means they can be used without the risk of disrupting embryo development. After completion of the preliminary stage of the study, in vivo tests with live animals and further studies were conducted using catheters made of PE and PU. In the case of both of these, material limitations did not occur, and it was possible to obtain a catheter of any diameter, even below 0.5 mm (unpublished data). The small diameter, flexibility and elasticity of these materials facilitated the introduction of the catheter bearing embryos into the uterus immediately after piercing the wall; the best embryo deposit depth achieved was 3–5 cm. After transfer of embryos using PE and PU catheters, a higher effectiveness was obtained than that shown by other authors. Among pig sows, the effectiveness of the laparoscopic ET method is from 10 to 60% [13,17,60,61,62,63,64,65]. Only Martinez, using a non-invasive embryo transfer method, obtained an effectiveness of more than 70% [20,66,67]. The high demonstrated effectiveness of embryo transfer was a confirmation of the appropriate choice of PE and PU as catheter materials. The appropriate quality and functionality of the catheter were ultimately confirmed in studies of the developmental potential of the transplanted embryos and in histological testing of the uterine tubes. After a 5-day incubation of the transferred embryos in the uterus, high developmental potential of the obtained blastocysts was demonstrated, with a high number of cell nuclei and a low apoptotic index. The same degree of cell nuclear defragmentation (13–16%) was confirmed as observed after surgical transplantation and 5-day embryo culture in vivo [68,69].

The histological examination indicated minimal trauma to the uterine tubes after introduction of the catheter, and these changes did not have an impact on the construction or functionality of the tubes. These results indicate the lack of a harmful impact of both the dedicated material and the proposed laparoscopic transfer procedure. The obtained results can also be successfully applied to other animal species. The high biocompatibility of the materials, the high quality of the catheters, and the ability to successfully perform embryo transfer in a difficult laparoscopic model provide the basis for trials in other animal species. This also opens the possibility of developing other tools for contact with embryos. The catheter is intended to be an important tool for minimally invasive embryo transfer with significantly reduced trauma compared to traditional surgical methods [65]. In the presented procedure, abdominal muscle wounds do not require suturing and are left to heal spontaneously, while single, simple sutures are placed on the skin. Atraumatic fallopian tube stabilization is also ensured. As a result, adhesions and other complications occurring after surgery are eliminated, and the risk of complications after the procedure is low [65].

## 5. Conclusions

In a comprehensive model of the study into the introduction of new materials in animal reproduction, the most important factor would seem to be the confirmation of the biocompatibility of the material in embryo cultures as available biomaterials with proven biocompatibility in relation to somatic cells may be highly toxic in relation to embryos. At the same time, it is essential to determine the material properties of such materials with regard to the possibility of freely forming the shape and diameter of the tubes while maintaining elasticity, durability, stability of the tubes, and a smooth surface. Among the 7 biomaterials tested, only polyethylene and polyurethane exhibited high biocompatibility and the material properties mentioned above. There is thus good indication for the introduction of these catheters for embryo transfer in animal reproduction biotechnology.

## Figures and Tables

**Table 1:** Verification of cytotoxicity of materials in somatic cell and embryo culture.

Item	Material								
	PTEF	PCL	PLLA/DBC	PU	PP	PLLA	PE	Control	Statistical Differences
Somatic cells—fibroblast survival, HS-5 cell line (%)	89.0%	96.0%	94.0%	90.0%	96.0%	91.0%	94.0%	100.0%	
Number of embryos in culture	15	17	20	15	22	22	15	15	
Number of dividing embryo	-	-	-	12	20	22	14	13	
Number of morulae	-	-	-	12	20	22	14	12	
% of morulae	-	-	-	80.0 ^A^	91.0 ^A,B^	100.0	93.0	80.0 ^B^	A: PPvsControl—*p* = 0.042 B: PLLAvsControl—*p* = 0.042
Number of blastocysts	-	-	-	12	20	20	12	10	
% of blastocysts	-	-	-	80.0	91.0	91.0	80.0	60.0	
Average number of cells/blastocyst	-	-	-	58.7 ^C,D^	30.7 ^C,E^	31.4 ^D,F^	57.8 ^E,F^	58.8	C: PUvsPP—*p* = 7.9 × 10^−15^ D: PUvsPLLA—*p* = 4.5 × 10^−20^ E: PPvsPE—*p* = 6.7 × 10^−15^ F: PLLAvsPE—*p* = 5.0 × 10^−20^
Average number of apoptotic cells/blastocyst (Apoptotic index; %)	-	-	-	8.3 ^G,H^ (14.2)	1.0 ^G,I^ (3.1)	1.3 ^H,J^ (3.6)	7.5 ^I,J^ (13.1)	6.8 (11.5)	G: PUvsPP—*p* = 8.4 × 10−^16^ H: PUvsPLLA—*p* = 6.1 × 10^−14^ I: PPvsPE—*p* = 1.2 × 10^−18^ J: PLLAvsPE—*p* = 9.7 × 10^−15^

**Table 2:** Assessment of the surface structure and surface roughness of the external and internal surfaces of the tubes.

Item		Group							Statistical Differences
		PE	PP	PLLA	PLLA/DBC	PCL	PTFE	PU	
Ra—external surface	Mean (±SD)	2.18 ^A,B,C,D,E^ ±0.3	3.34 ^A,F^ ±0.9	5.24 ^B,G^ ±1.4	4.32 ^C,H^ ±0.9	2.62 ^D^ ±0.3	2.99 ^E^ ±0.3	2.35 ^F,G,H^ ±0.4	A: PEvsPP—*p* = 0.00036 B: PEvsPLLA—*p* = 1.5 × 10^−7^ C: PEvsPLLA/DBC—*p* = 9.5 × 10^−8^ D: PEvsPCL—*p* = 0.00419 E: PEvsPTFE—*p* = 0.00001 F: PPvsPU—*p* = 0.00219 G: PLLAvsPU—*p* = 4.8 × 10^−7^ H: PLLA/DBCvsPU—*p* = 4.0 × 10^−7^
Ra—internal surface	Mean (±SD)	2.04 ^A,B,C,D,E^ ±0.2	3.39 ^A,F^ ±0.6	4.73 ^B,G^ ±0.9	3.97 ^C,H^ ±0.7	2.35 ^D^ ±0.3	2.64 ^E,I^ ±0.5	2.13 ^F,G,H,I^ ±0.5	A: PEvsPP—*p* = 4.3 × 10^−7^ B: PEvsPLLA—*p* = 1.0 × 10^−9^ C: PEvsPLLA/DBC—*p* = 1.6 × 10^−8^ D: PEvsPCL—*p* = 0.0091 E: PEvsPTFE—*p* = 0.00086 F: PPvsPU—*p* = 0.00002 G: PLLAvsPU—*p* = 1.2 × 10^−8^ H: PLLA/DBCvsPU—*p* = 4.0 × 10^−7^ I: PTFEvsPU—*p* = 0.02058
Rt—external surface	Mean (±SD)	37.94 ^A,B,a^ ±17.4	45.87 ^b^ ±14.8	69.27 ^c^ ±24.9	58.05 ^A,C,d^ ±20.9	37.94 ^B,e^ ±10.4	40.70 ±16.4	39.36 ^C,f^ ±14.1	A: PEvsPLLA/DBC—*p* = 0.0017 B: PEvsPCL—*p* = 0.018 C: PLLA/DBCvsPU—*p* = 0.018
Rt—internal surface	Mean (±SD)	17.05 ^A,B,C,D,a^ ±8.9	26.40 ^b^ ±11.5	47.78 ^A,E,c^ ±13.54	37.27 ^B,F,d^ ±12.22	17.14 ^C,e^ ±7.87	30.50 ±13.87	19.31 ^D,E,F,f^ ±10.88	A: PEvsPLLA—*p* = 0.0304 B: PEvsPLLA/DBC—*p* = 9.8 × 10^−7^ C: PEvsPCL—*p* = 0.0001 D: PEvsPU—*p* = 0.0082 E: PLLAvsPU—*p* = 0.00001 F: PLLA/DBCvsPU—*p* = 0.001
Rz—external surface	Mean (±SD)	14.20 ^A,B,C^ ±5.3	20.47 ^D^ ±5.6	29.37 ^A,E^ ±11.1	25.6 ^B,F^ ±10.7	15.56 ^C^ ±5.0	17.21 ±5.9	14.76 ^D,E,F^ ±5.4	A: PEvsPLLA—*p* = 0.0102 B: PEvsPLLA/DBC—*p* = 0.0003 C: PEvsPCL—*p* = 0.0032 D: PPvsPU—*p* = 0.0182 E: PLLAvsPU—*p* = 0.0005 F: PLLA/DBCvsPU—*p* = 0.0047
Rz—internal surface	Mean (±SD)	13.51 ^A,B^ ±5.9	18.24 ±6.0	28.87 ^A,C^ ±9.5	23.76 ^B,D^ ±8.1	14.97 ±4.0	16.21 ^E^ ±4.4	14.83 ^C,D^ ±3.1	A: PEvsPLLA—*p* = 0.0001 B: PEvsPLLA/DBC—*p* = 0.0019 C: PLLAvsPU—*p* = 0.0001 E: D: PLLA/DBCvsPU—*p* = 0.0018

SD—standard deviation. Ra—arithmetic mean deviation of the roughness profile, Rt—maximum height between the highest peak and the lowest valley, Rz—height of the roughness profile assessed according to ten points (5 highest peaks and 5 lowest valleys). Statistical differences between external and internal surfaces of the catheter: a—Rt external vs internal PE *p* = 0.0010; b—Rt external vs internal PP—*p* = 0.0016; c—Rt external vs. internal PLLA—*p* = 0.015475; d—Rt external vs. internal PLLA/DBC—*p* = 0.007171; e—Rt external vs. internal PCL—*p* = 0.000016; f—Rt external vs. internal PU—*p* = 0.000791.

**Table 3:** Study of tube geometry and stability of shape.

Item	Group							Statistical Differences
	PE	PP	PLLA	PLLA /DBC	PCL	PU	PTFE	
Outer diameter [mm]	1.06 ^A,B,C,D,E,F^ ±0.09	1.83 ^A^ ±0.15	3.25 ^B^ ±0.52	3.40 ^C^ ±0.7	1.23 ^D^ ±0.16	1.64 ^E^ ±0.28	1.31 ^F^ ±0.19	A: PEvsPP—*p* = 3.1 × 10^−14^ B: PEvsPLLA—*p* = 3.0 × 10^−15^ C: PEvsPLLA/DBC—*p* = 3.6 × 10^−13^ D: PEvsPCL—*p* = 0.005443 E: PEvsPU—*p* = 1.9 × 10^−7^ F: PEvsPTFE—*p* = 0.000721
Δ Outer diameter (%)	14%	14%	29%	31%	19%	28%	26%	
Outer surface area [mm^2^]	0.88 ^A,B,C,D,E^ ±0.14	2.65 ^A^ ±0.4	8.49 ^B^ ±2.84	9.43 ^C,F^ ±3.6	1.22 ^D,F^ ±0.32	2.17 ^E^ ±0.77	1.37 ±0.4	A: PEvsPP—*p* = 0.000379 B: PEvsPCL—*p* = 2.1 × 10^−9^ C: PEvsPLLA/DBC—*p* = 1.4 × 10^−8^ D: PEvsPCL—*p* = 8.9 × 10^−8^ E: PEvsPU—*p* = 0.000018 F: PLLA/DBCvsPCL—*p* = 1.5 × 10^−9^
Δ Outer surface area (%)	27% ^A,B^	15% ^A^	41% ^B^	56%	38%	52%	52%	A: PEvsPP—*p* = 0.049 B: PEvsPLLA—*p* = 0.010
Inner diameter [mm]	0.77 ^A,B^ ±0.05	1.4 ±0.18	2.50 ^A^ ±0.38	2.75 ^B^ ±0.67	0.99 ±0.16	1.0 ±0.26	0.86 ±0.13	A: PEvsPLLA—*p* = 8.9 × 10^−12^ B: PEvsPLLA/DBC—*p* = 1.1 × 10^−8^
Δ Inner diameter (%)	10% ^A,B^	48%	56% ^A^	39% ^B^	15%	45%	19%	A: PEvsPLLA—*p* = 0.0014 B: PEvsPLLA/DBC—*p* = 0.0012
Inner surface area [mm^2^]	0.47 ^A,B,C,D,E^ ±0.06	1.54 ^A^ ±0.38	5.02 ^B^ ±1.63	6.25 ^C,F^ ±2.75	0.8 ^D,F,G^ ±0.25	1.04 ^E,G^ ±0.55	0.6 ±0.18	A: PEvsPP—*p* = 2.9 × 10^−10^ B: PEvsPLLA—*p* = 2.5 × 10^−11^ C: PEvsPLLA/DBC—*p* = 9.9 × 10^−9^ D: PEvsPCL—*p* = 0.0022 E: PEvsPU—*p* = 9.9 × 10^−9^ F: PLLA/DBCvsPCL—*p* = 2.5 × 10^−8^ G: PCLvsPU—*p* = 0.021
Δ Inner surface area (%)	19% ^A^	29% ^A^	45% ^B^	70% ^B^	51%	37%	50%	A: PEvsPP—*p* = 0.025 B: PLLAvsPLLA/PLLA/DBC—*p* = 0.017
Wall thickness [mm]	0.14 ^A,B,C,D^ ±0.03	0.22 ±0.03	0.37 ^A^ ±0.19	0.32 ±0.07	0.9 ^B^ ±0.2	0.26 ^C^ ±0.06	0.22 ^D^ ±0.05	A: PEvsPLLA—*p* = 1.2 × 10^−7^ B: PEvsPCL—*p* = 0.000038 C: PEvsPU—*p* = 0.0000028 D: PEvsPTFE—*p* = 0.0000003
Δ Wall thickness (%)	44% ^A,B^	50%	90% ^A^	60%	90% ^B^	62%	67%	A: PEvsPLLA—*p* = 0.00061 B: PEvsPCL—*p* = 0.00023

Δ—changes.

**Table 4:** The effectiveness of laparoscopic embryo transfer and the developmental potential of transplanted embryos.

Item	Group		
	PE (n = 20)	PU (n = 10)	Control
The effectiveness of laparoscopic embryo transfer			
Number of embryos transferred/recipient	28.0	27.3	-
Number of pregnancies achieved	15	7	-
Percentage of pregnancies achieved	75.0%	70.0%	-
Number of piglets born/gilt	10.4	10.0	-
Piglets born alive/gilt (%)	9.4 (90.0%)	9.1 (91.0%)	-
Piglets weaned/gilt (%)	9.0 (86.0%)	8.6 (85.3%)	-
The developmental potential of transplanted embryos			
Item	PE (n = 4)	PU (n = 4)	Control
Number transplanted embryos	64	60	5
Number of washed blastocysts	7	9	5
Total number of nuclei/blastocyst	54.86 ± 2.41	58.67 ± 2.15	58.00 ± 6.82
Number of apoptotic nuclei/blastocyst	8.00 ± 1.91	8.3 ± 1.91	8.23 ± 2.28
Apoptotic index (AI)F (%)	14.5 ± 0.03	14.2 ± 0.05	14.5 ± 0.05

**Table 5:** The results of histological examination of the uterine tubes after embryo transplantation with the PU, PE catheter and the control uterine tubes.

Item	PE (n = 4)	PU (n = 4)	Control (n = 8)	Statistical Difference
Preserved lumen of the fallopian tube	3	3	3	
Inflammatory infiltration	0.2	0.2	0.3	
Granulocyte infiltration in the lumen of the fallopian tube	0.25	0.4	0.0	
Granulocyte infiltration in the wall of the fallopian tube	0.5 ^A^	0.4	0.0 ^A^	A—*p* = 0.039
Purulent changes of the fallopian tube	0.2	0.0	0.0	
Purulent changes in the fallopian tube	0.0	0.0	0.0	

## Data Availability

Dataset available on request from the authors.
